# Signals of recent spatial expansions in the grey mouse lemur (*Microcebus murinus*)

**DOI:** 10.1186/1471-2148-10-105

**Published:** 2010-04-22

**Authors:** Nicole Schneider, Lounès Chikhi, Mathias Currat, Ute Radespiel

**Affiliations:** 1 Institute of Zoology, University of Veterinary Medicine Hannover, Bünteweg 17, 30559 Hannover, Germany; 2 CNRS, EDB (Laboratoire Evolution et Diversité Biologique), UMR CNRS/UPS 5174, F-31062 Toulouse, France; 3 Université de Toulouse, UPS, EDB (Laboratoire Evolution et Diversité Biologique), Bâtiment 4R3 b2, 118 Route de Narbonne, F-31062 Toulouse, France; 4 Instituto Gulbenkian de Ciência, Rua da Quinta Grande 6, 2780-156 Oeiras, Portugal; 5 Laboratory of Anthropology, Genetics and Peopling history (AGP), Department of Anthropology and Ecology, University of Geneva, 12 rue Gustave-Revilliod, 1211 Genève 4, Switzerland

## Abstract

**Background:**

Pleistocene events have shaped the phylogeography of many taxa worldwide. Their genetic signatures in tropical species have been much less explored than in those living in temperate regions. We analysed the genetic structure of a Malagasy primate species, a mouse lemur with a wide distribution (*M. murinus)*, in order to investigate such phylogeographic processes on a large tropical island. We also evaluated the effects of anthropogenic pressures (fragmentation/deforestation) and natural features (geographic distance, rivers) on genetic structure in order to complement our understanding of past and present processes of genetic differentiation.

**Results:**

The analysis of the mitochondrial D-loop sequences of 195 samples from 15 study sites (10 from a continuous forest and five from isolated forest fragments) from two adjacent Inter-River-Systems (IRSs) revealed that forest fragmentation and the river restrict gene flow, thereby leading to an increased genetic differentiation between populations beyond the effect of isolation-by-distance. Demographic simulations detected signals of two successive spatial expansions that could be preliminarily dated to the late Pleistocene and early Holocene. The haplotype network revealed geographic structure and showed deep molecular divergences within and between the IRSs that would be congruent with a two-step colonization scenario.

**Conclusions:**

This study supports the hypothesis of a relatively recent spatial expansion of the grey mouse lemur in northwestern Madagascar, which may also explain why this taxon, in contrast to its congeners, has not yet undergone allopatric speciation in the studied area and possibly across its presently wide range.

## Background

Pleistocene events have shaped the phylogeography of many taxa worldwide [1]. Particular emphasis has been put on these dynamics in temperate regions [2]. Tropical biomes have also been shown to have undergone complex ecological dynamics following worldwide Pleistocene climate changes [3], but their genetic signatures in present-day natural populations are much less explored. The genetic structure of natural populations is not only shaped by these ancient processes but also by ongoing natural (e.g. migration, genetic drift) and more recent anthropogenic factors [4].

One model region for the study of the combined effects of ancient and recent factors is Madagascar. This island has been isolated from all other landmasses during the last 80 million years (My) and different vertebrate groups have undergone complex phylogeographic histories [5]. Moreover, Madagascar has been strongly affected by anthropogenic disturbances during the last 2000 years following the arrival of humans on the island [6,7]. Approximately 90% of the original vegetation is believed to have already disappeared ([8], but see [9] for less extreme estimates) and most forests are now heavily fragmented [10,11]. This poses a particular threat to forest-dependent species, since barriers such as novel savannahs can significantly modify their genetic structure [12]. Such barriers can notably reduce gene flow between populations which can lead to a loss of alleles, an increase in homozygosity and inbreeding through isolation [13].

Lemurs are an endemic mammal group on Madagascar. Within lemurs, mouse lemurs (*Microcebus *spp.) form an exceptionally diverse genus. The number of described *Microcebus *species has increased from four to 18 in recent years (overview in [14,15]) due to largely increased sampling efforts and to the application of phylogenetic analyses of DNA sequences. Mouse lemurs are small, nocturnal, solitary foragers that inhabit a large variety of forest habitats with no more than two species co-occurring in a given area (see [16] for a review).

The high species diversity of lemurs in general and of mouse lemurs in particular has been explained as a joint effect of topographic barriers (large rivers, mountains) and/or Pleistocene climatic and vegetation changes [17-19]. Recent studies indicate that most large rivers act as genetic barriers for mouse lemurs [19,20] and it has been suggested that a long-term separation between adjacent Inter-River-Systems (IRSs) promoted speciation in this clade [19]. This process may explain the very limited distributions of many species.

In contrast to most other lemur species, the grey mouse lemur (*M. murinus*) has a wide distribution as it inhabits the dry deciduous forests from southern to northwestern Madagascar [14]. The apparent lack of speciation events within this taxon could be the result of a lower speciation rate, a higher migratory potential, or a relatively recent expansion of this species into the western IRSs of Madagascar. The latter hypothesis is supported by three lines of evidence and shall be further investigated in this study: *i) *In a recent study Kappeler et al. [21] estimated the age of the *M. murinus *clade to about 1.4 My, which sets an upper Pleistocene limit for its expansion; *ii) M. murinus *possesses an ecological preference for dry habitats [22] which are typical for southern Madagascar; *iii) M. murinus *is the closest relative of *M. griseorufus *from southern Madagascar [19,23] which suggests that this expansion may have originated in southern Madagascar. This geographic setting would indeed suggest a late Pleistocene or even postglacial colonization of western to northwestern Madagascar by the grey mouse lemur.

**Figure 1:**
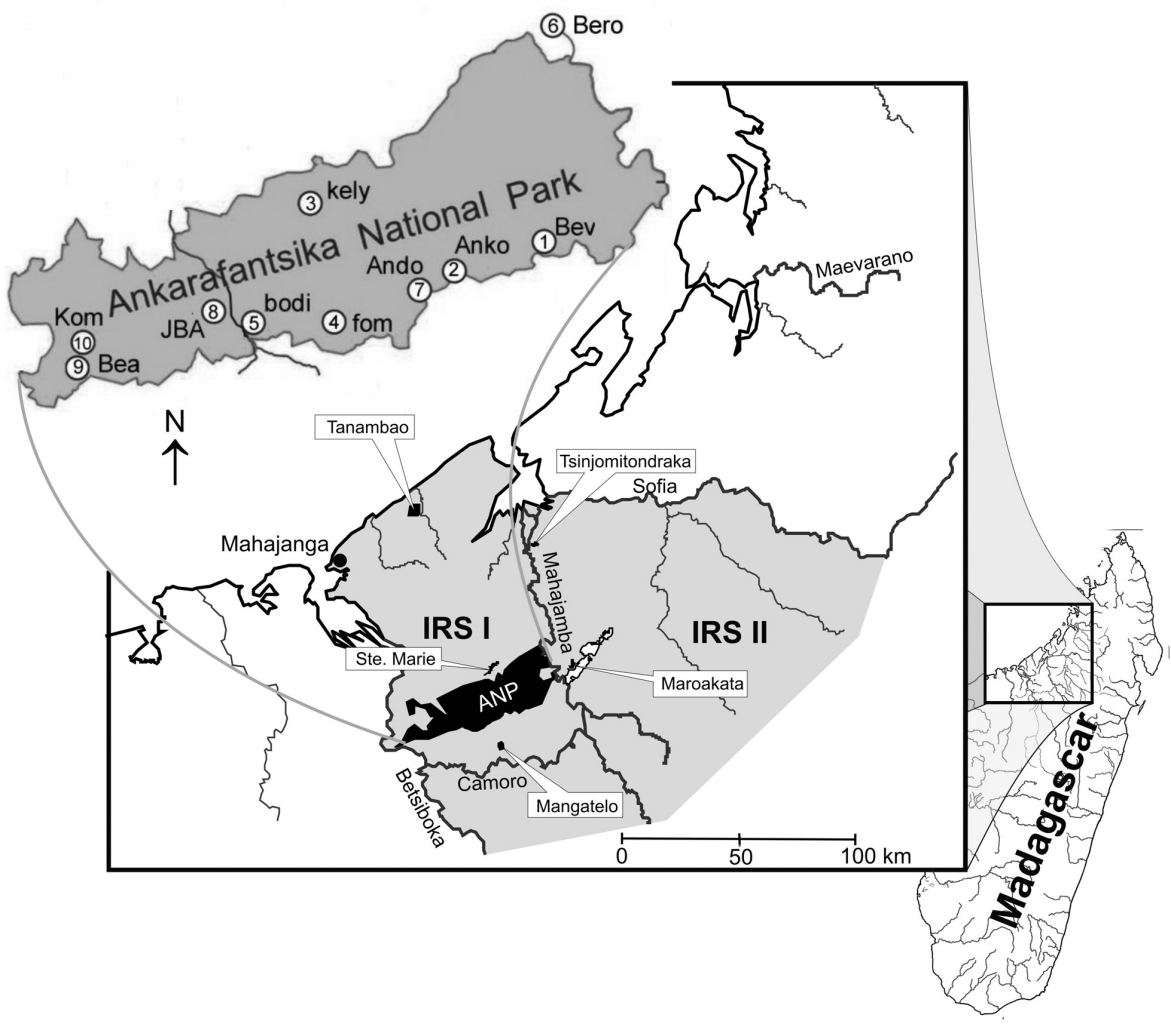
**Map of northwestern Madagascar showing the sampling sites within the two Inter-River-Systems (IRS1 and IRS2)**. The studied forest fragments are indicated with the labelling boxes. A close up of the Ankarafantsika National Park (Ank NP), showing the location of the ten study sites within the park, is indicated in the upper left corner.

Grey mouse lemurs occur in partial sympatry with the golden-brown mouse lemur (*M. ravelobensis*) and with the Bongolava mouse lemur (*M. bongolavensis*) in two adjacent IRSs at their northern distribution limit ([19], Figure 1). The effect of natural and anthropogenic factors on population structure and genetic diversity of *M. ravelobensis *and *M. bongolavensis *has been studied in some detail using mtDNA [24] and microsatellites [12,25]. These studies revealed a negative influence of forest fragmentation on genetic diversity [24] and showed an effect of isolation-by-distance for *M. ravelobensis *[12,25]. Moreover, large rivers and savannahs were shown to reduce or even prevent gene-flow between populations and have generated genetic structure [24,25].

This study aims to assess the effects of phylogeographic processes, anthropogenic pressures (fragmentation/deforestation), and natural features (geographic distance, rivers) on the genetic structure of the grey mouse lemur at its northern distribution edge. Populations in this area are predicted to show signs of a Pleistocene spatial expansion from the likely southern origin towards the northwestern IRSs. Spatial simulations were used to identify the expansion model which fitted best to the patterns of molecular diversity observed in extant *M. murinus *populations. The results are compared to previously published data on *M. ravelobensis*, which may have arrived before. By means of spatial simulations, we could detect signals of two successive spatial expansions of *M. murinus *in northwestern Madagascar which could be preliminarily dated to the late Pleistocene and the early Holocene.

## Results

### Genetic diversity

A total of 367 monomorphic (81.9%) and 81 polymorphic positions (18.1%) were identified in the D-loop sequences (455 bp) of the 195 individuals. A total of 47 different haplotypes were found which differed in 1-37 positions in pairwise comparisons.

The genetic diversity of sites varied in the ANP, as well as among the isolated forest fragments (Table 1). Three sites exhibited no genetic variation. These were two sites with small sample sizes in the ANP (Ambodimanga, Komandria) and one forest fragment with a large sample size (Maroakata, in IRS2). However, we found no overall significant influence of sample size on the number of haplotypes or on haplotype diversity (no. of haplotypes: R^2 ^= 0.104, F(1,13) = 1.507, n.s.; haplotype diversity: R^2 ^= 0.002, F(1,13) = 0.028, n.s.).

**Table 1:** Fragment size, sample size, genetic diversity and results of neutrality tests for the different sites

System	Site name (abbreviation in bold)	Fragment size (km^2^)	Sample size	H(n)	Hd	AR_(r)_	Nd (π)	Tajima's *D*	Fu's *Fs*
	**Bev**azaha	1040	11	5	0.709	2.089	0.013	-0.415	2.654
	
	**Anko**ririka	1040	23	7	0.767	2.294	0.019	-0.548	5.226
	
	Ambanja**kely**	1040	14	7	0.846	2.739	0.032	0.590	4.485
	
	Ando**fom**bobe	1040	21	7	0.8	2.444	0.04	1.685	10.071
	
IRS 1	Am**bodi**manga	1040	5	1	0	0	0	-	-
	
**(ANP)**	**Bero**nono	1040	5	2	0.4	1	0.01	-1.205*	4.937
	
	**Ando**harano	1040	18	5	0.556	1.535	0.019	-0.781	6.924
	
	**JBA**	1040	12	7	0.833	2.739	0.018	-1.204	1.443
	
	**Bea**lana	1040	3	3	1	-	0.015	0.000	0.703
	
	**Kom**andria	1040	3	1	0	-	0	-	-

	**S^te ^M**arie	5.4	11	4	0.673	1.786	0.03	2.316	8.061
	
IRS 1	Manga**telo**	17.7	27	2	0.074	0.185	0.0003	-1.512*	-0.299
	
**(IFFs)**	**Tan**ambao	36.4	10	6	0.844	2.754	0.014	-1.329	1.022

IRS 2	Maroak**ata**	2	21	1	0	0	0	-	-
	
**(IFFs)**	**Tsin**jomitondraka	40.8	11	3	0.564	1.333	0.003	0.336	1.435

H(n): number of haplotypes; Hd: haplotype diversity; AR_(r)_: Allelic richness, corrected for sample size; Nd (π): nucleotide diversity; IFF: isolated forest fragment; ANP: National Park; -: values could not be calculated due to the lack of polymorphism (*D *or *Fs*) or small sample size (AR_(r)_); *: p < 0.02.

In general, the ANP samples showed the highest genetic diversity, in particular sites with larger sample sizes (*n *> 5). The four samples with most haplotypes (*n *= 7) were located within the ANP. The three ANP samples with only one or two haplotypes were also the smallest samples with three or five individuals, respectively. Among the isolated fragments, Tanambao possessed the highest number of haplotypes (*n *= 6), while the other sites were less diverse. When comparing haplotype diversity, allelic richness or nucleotide diversity statistically between the sites in the ANP and the isolated forest fragments, however, no significant difference could be revealed (Mann-Whitney-U: Hd: U = 19, n_1 _= 10, n_2 _= 5, n.s.; AR_(r)_: U = 14.5, n_1 _= 8, n_2 _= 5, n.s.; Nd: U = 17; n_1 _= 10, n_2 _= 5, n.s.). This lack of statistical evidence could be due to a lack of power given the small overall number of study sites and some variability among fragments and among ANP sites.

### Demographic history

Table 1 shows the results of Tajima's *D *and Fu's *Fs *tests. Six out of 12 values of Tajima's *D *were negative, two of them significantly (Beronono, Mangatelo), while none of the populations showed significantly positive *D *values. Similarly, no Fu's *Fs *values were significant (Table 1). No tests were carried out for Ambodimanga, Komandria and Maroakata due to their lack of polymorphism.

**Figure 2:**
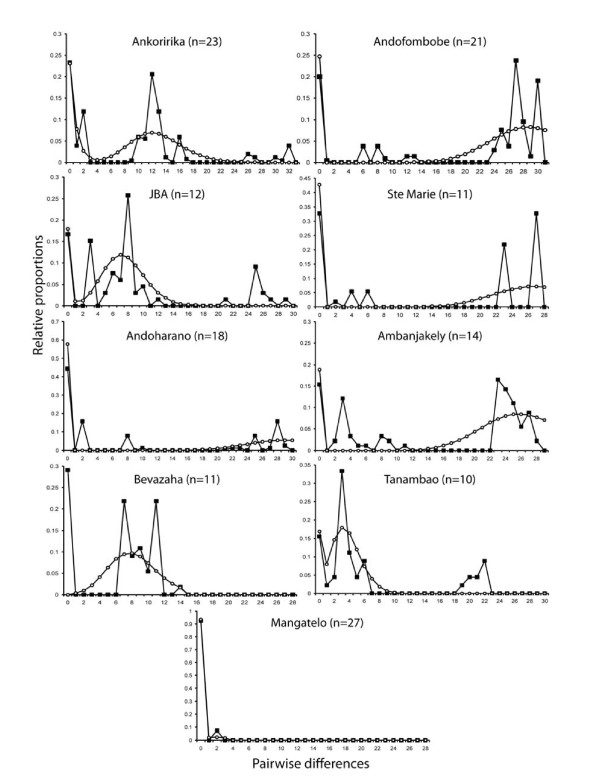
**Mismatch distributions observed in samples from the IRS1 with a minimum size of 10 individuals**. Open circles: simulated mismatch distribution, black squares: observed mismatch distribution.

**Figure 3:**
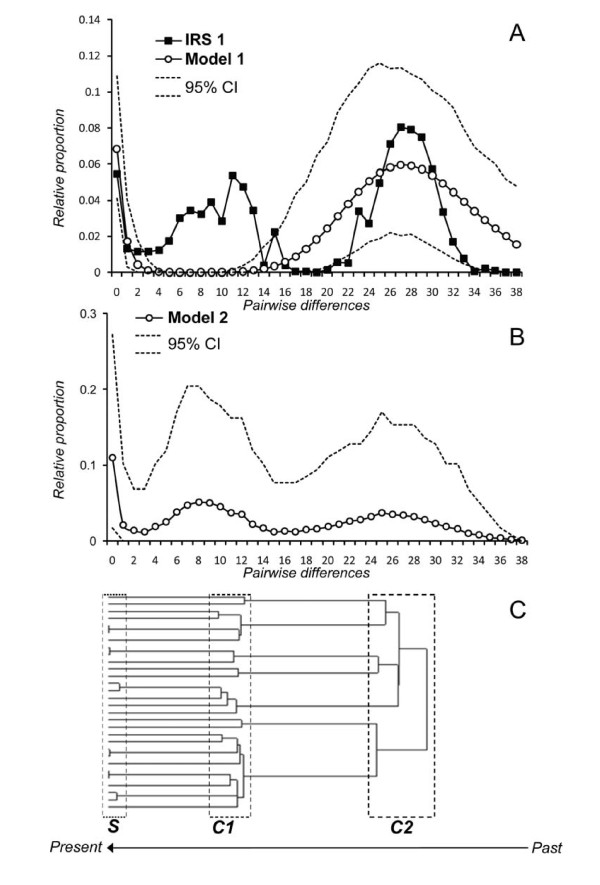
**Observed and simulated *mismatch distributions***. A) *Mismatch distributions *calculated for the total sample of IRS1 (black squares, n = 163) as well as for *Model 1 *(one spatial expansion) (open circles) and its 95% confidence interval *(dotted lines)*. The Y axis stands for the average probability that two DNA sequences differ at a given number of sites represented on the X axis. B) *Mismatch distribution *obtained by simulation of *Model 2 *(two successive spatial expansions). The solid line shows the average mismatch distribution obtained from 1,000 simulations of the coalescent of 30 genes drawn in a single deme after two successive spatial expansions have occurred 10 and 30 mutational units of time ago. Dotted lines delimit an empirical 95% confidence interval for the *mismatch distribution*. C) Typical gene genealogies obtained for *Model 2*. Three main phases of coalescence (S, C1 and C2) are translated into the three modes of the *mismatch distribution *(see text).

The *mismatch distributions *were not significantly different from those expected under one spatial expansion (*Model 1*) for any of the samples (Table 2). The visual inspection of the distributions, however, revealed three distinct peaks, despite the relative raggedness of the *mismatches *for single samples (Figure 2). The presence of three modes was particularly clear when all samples of IRS1 were grouped together to increase sample size (IRS1 total, Figure 3a). The first mode was located at zero pairwise differences and was visible in all samples and in the expected distribution generated by the simulation of one spatial expansion (Figure 3a and Figure 2). The second mode ranged from 5 to 15 pairwise differences and the third from 23 to 33 pairwise differences. Only two of the three modes observed in the data were produced in simulations of one spatial expansion (*Model 1*), either the first and the second or the first and the third (open circles in Figure 3a, Figure 2). This pattern indicates that a more complex model is necessary to better fit the data, suggesting that two spatial expansions separated by a period of demographic contraction may be more accurate (*Model 2*). As no specific statistical test is available to determine if a *mismatch distribution *fits the *Model 2*, we tested this hypothesis using simulations.

**Table 2:** Results of the *mismatch distributions*, tested for a single spatial expansion (*Model 1*, 1000 bootstraps)

Sample	Mismatch observed mean	Mismatch observed variance	Tau	Significance
**Bevazaha**	6.436	19.695	9.159	n.s.

**Ankoririka**	9.348	72.736	11.278	n.s.

**Ambanjakely**	15.626	121.37	26.227	n.s.

**Andofombobe**	19.786	139.758	29.171	n.s.

**Andoharano**	9.261	141.747	29.602	n.s.

**JBA**	8.682	66.897	7.301	n.s.

**S^te ^Marie**	14.436	151.251	27.232	n.s.

**Mangatelo**	0.148	0.275	2.327	n.s.

**Tanambao**	6.511	56.574	3.676	n.s.

**Tsinjomitondraka**	1.491	2.255	3.109	n.s.

Tau: age estimator for the expansion (Tau = 2 *Tμ*) [45].

**Figure 4:**
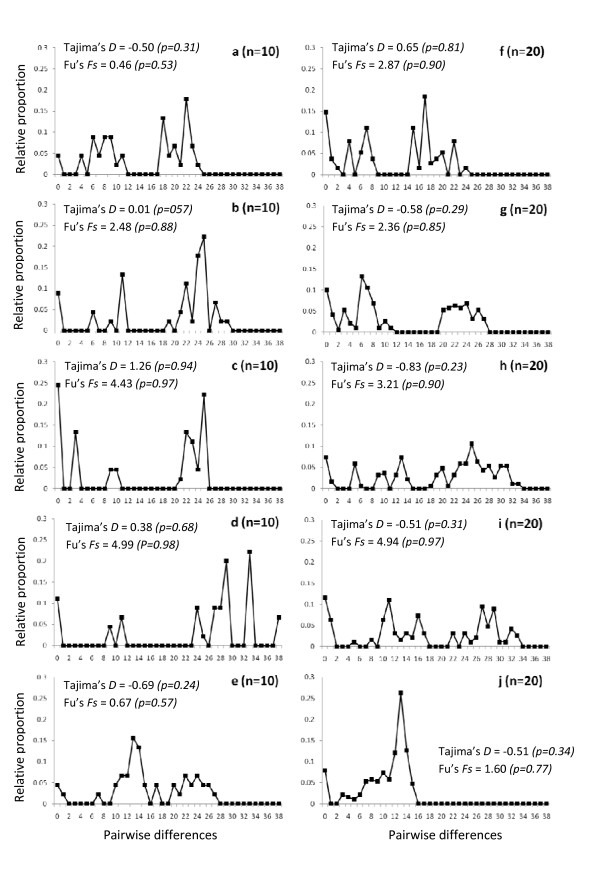
**Mismatch distributions obtained for single realizations of the coalescent after two consecutive spatial expansions**. The parameters are specified in the Materials and methods section. Tajima's *D* and Fu's *F*s values are given. Left column (graphs a-e): sample size = 10 mtDNA sequences; Right column (graphs f-j): sample size = 20 mtDNA sequences.

As shown in Figure 3b, the two simulated successive expansions did indeed produce three modes which can be explained by three main phases of coalescence. Going backward in time these phases can be described using the terminology that Wakeley [26,27] used to describe the coalescent in structured models: They are called the "scattering" and the "collecting" phases (*S *and *C*). *S *is characterized by a series of recent coalescent events which correspond to individuals in the sample that are related (i.e., they have recent ancestors, and so do their alleles). The two older phases (*C1 *and *C2*) correspond to the "collecting phases" which occur at the level of the metapopulation, and in our case during the spatial contraction (going backward in time). They correspond to the alleles whose common ancestor can be traced to the time of the range expansion (going forward in time). In a widely distributed but randomly mating population, coalescent events are rare and randomly distributed in time (according to the coalescent theory), but during a range expansion from one region, many coalescent events occur around the time of the expansion because individuals are restricted to a smaller geographic area. In the simulated *Model 2*, the three main periods of coalescence translate into three modes in the *mismatch distribution*. In the gene genealogy (Figure 3b), we thus see that if one population expands during two separate periods, we can expect to observe two collecting phases, which will generate *mismatch distributions *similar to those observed in the dataset (Figure 3a). Note that when looking at single simulations instead of the average distribution, the three modes are not always clearly visible together (Figure 4). This result fits well with our observed *mismatch distributions *for single samples (Figure 2).

### Genetic differentiation between populations

Pairwise Φ_ST _values ranged widely from 0 to 1, but most values (83 out of 91) were above 0.2 and only seven were not significant (Additional file 1: Table S1). Therefore, the sites can be generally regarded as genetically differentiated from each other. Genetic (Φ_ST _values) and geographic distances (*km*) were significantly and positively correlated in the IRS1 (Mantel test, r_p _= 0.487, n = 13, p < 0.001) suggesting the existence of isolation-by-distance within IRS1.

### Influence of the Mahajamba River

The importance of the Mahajamba River as a barrier to gene flow between IRS1 and IRS2 was tested with an AMOVA and with permutation tests. All variance components and fixation indices were large and significantly different from zero. The highest proportion of the molecular variance (41.2%) was explained by the variations between sites within each IRS while 32.8% is explained by the river (the remaining 26.0% of the molecular variance is explained by the variation within sites). These values should be interpreted with care, however, since we only had two samples in IRS2 which are almost fixed for very different haplotypes. The permutation tests showed that average Φ_ST _values across the Mahajamba river were significantly higher than those between samples from the same side of the river (averages: 0.87 vs. 0.55, p < 0.001), but we note that some pairwise Φ_ST _values between populations from the same side of the river were also very high (seven out of 34 were greater than 0.87, the across river average).

### Influence of forest fragmentation

**Figure 5:**
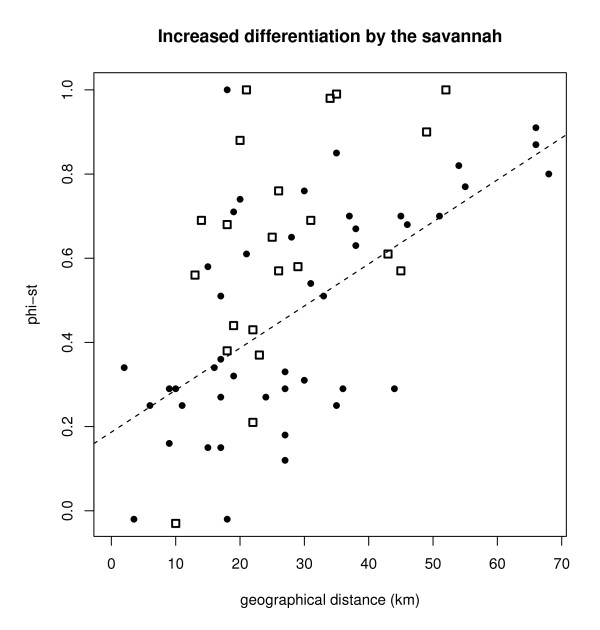
**Relationship between geographical distance and Φ_ST_-values**. Black circles: pairs of park populations (n = 45), open squares: pairs of populations that are separated by stretches of savannah (n = 21), dashed line: regression line for pairs of ANP populations.

Pairwise Φ_ST _values were significantly higher between populations separated by a savannah (*n *= 21) than between populations separated by a "continuous" forest habitat (i.e., within the ANP, *n *= 45; means: 0.66 vs. 0.47, p < 0.01, Figure 5). This was confirmed by the analysis of the residuals, which were significantly higher for samples separated by savannah stretches. This suggests that populations separated by savannah stretches are more prone to drift than those within the ANP, probably due to the more limited size of the fragments they live in.

### Genetic structure by haplotype network

**Figure 6:**
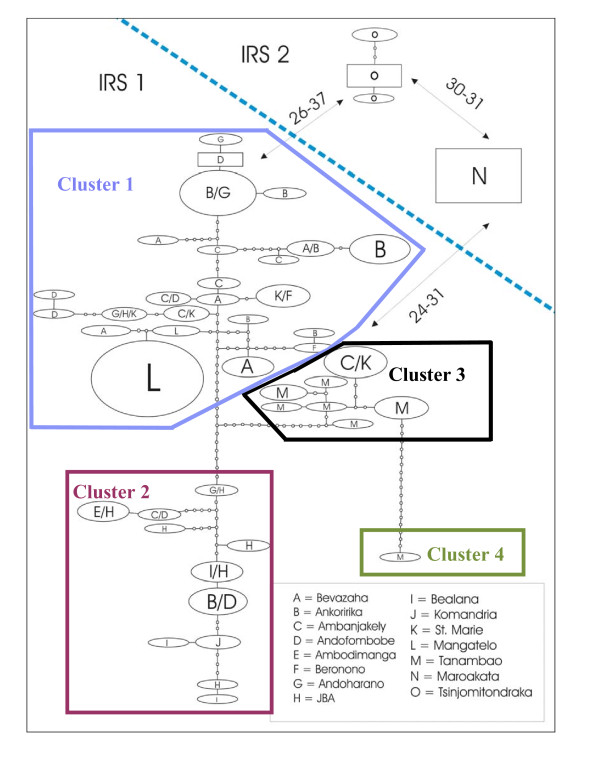
**Haplotype networks in IRS1 and IRS2**. The size of ovals and squares is proportional to the number of individuals that share a haplotype. The haplotypes with the highest outgroup probability are displayed as a square, the other haplotypes are displayed as ovals [54]. Each node represents a mutation step between haplotypes. The numbers of mutation steps between the networks of the IRS2 and the network of the IRS1 are provided (see arrows). The haplotypes of IRS1 belong to four different clusters, separated by at least ten mutation steps.

Thirteen of 47 haplotypes (27.7%) were shared among several sites (Figure 6). These sites were all located within the ANP with the exception of the S^te ^Marie fragment which shared all its haplotypes with different ANP populations. S^te ^Marie, however, is located only 3 km from the ANP. Within the ANP, haplotype sharing mostly occurred between neighbouring sites. The remaining four forest fragments had only private haplotypes.

**Figure 7:**
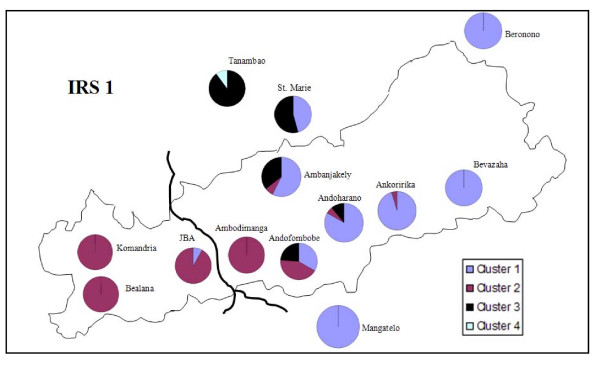
**Schematic map of the sampling populations in the IRS1 with the outline of the ANP as a thin black line**. The proportion of individuals of each site belonging to clusters 1-4 (see Figure 6 for the delineation of the clusters) are indicated in site-specific pie-charts.

The network analysis revealed three distinct sets of haplotypes which were separated by more than 23 mutation steps from each other (Figure 6). The first set was within IRS1. It contained 43 different haplotypes which showed 1-37 pairwise differences, and could be grouped into four clusters separated by more than 10 mutational steps. Even though the most divergent haplotypes are very different, there are many intermediate haplotypes. The other haplotypes were located within the IRS2 and were separated by at least 30 mutation steps from each other and by 24-26 mutations from the closest IRS1 haplotype. One haplotype was limited to Maroakata and the other three to Tsinjomitondraka. In contrast, the spatial structure was not as obvious within the IRS1, as haplotypes observed within one site were not always more similar to each other than to haplotypes from other sites. However, a spatial structure was revealed after mapping the representation of the four clusters in IRS1 (Figure 7). The sites in the ANP could be partitioned into a homogenous western and eastern division, respectively, and a zone of heterogeneous composition in the centre. The four westernmost sites in the ANP (Komandria, Bealana, JBA, Ambodimanga) mainly contained haplotypes of cluster 2, whereas the four eastern sites in the ANP (Beronono, Bevazaha, Ankoririka, Andoharano) and the fragment Mangatelo mainly contained haplotypes of cluster 1. Haplotypes of cluster 3 were only found in three heterogeneous central park populations (Ambanjakely, Andoharano, Andofombobe) and the two fragments S^te ^Marie and Tanambao. Finally, cluster 4 which consists of one haplotype, was only present in the northernmost fragment Tanambao.

## Discussion

This study provides evidence for two successive spatial expansions of *M. murinus *in northwestern Madagascar (see below) but could also demonstrate some effects of forest fragmentation, geographic distance and the large river Mahajamba on the genetic structure of this widely distributed mouse lemur species. These anthropogenic and environmental effects shall be discussed first, before we move to the phylogeographic processes.

### Effects of forest fragmentation and environmental features on genetic diversity and genetic structure

Our results suggest that forest fragmentation significantly increased genetic drift in the isolated fragments. However, even though the genetic diversity of samples from the ANP was generally high, the difference with isolated fragments was not significant. This suggests that a larger dataset (both in terms of number of study sites and within-site sample sizes) would be required to draw stronger conclusions about the effect of forest fragmentation on the diversity of *M. murinus*. When compared to the sympatric *M. ravelobensis*, the number of haplotypes and the haplotype diversity per site were not significantly different (H_ANP_: 3-8, H_IFF_: 1-8, Hd_ANP_: 0.542-0.880, Hd_IFF_: 0-0.656, [28], four comparisons with MWU-test: n.s.). Therefore, the effect of fragmentation on genetic diversity can be assumed to be similar in both species.

Besides affecting the genetic diversity within populations, fragmentation also increased the genetic differentiation between them beyond the effects of isolation-by-distance which was also detected. The average Φ_ST _values between sites isolated by savannah were significantly higher than those between sites from the ANP (i.e. continuous forest) despite comparable geographic distances. Furthermore, haplotype sharing was typically not observed between isolated fragments but only between sites in the ANP. The only exception was S^te ^Marie that shared its four haplotypes with different Park populations. It must be emphasized, though, that S^te ^Marie is separated from the ANP only by a 3 km stretch of savannah. Surprisingly, a previous study on *M. ravelobensis *in S^te ^Marie did not find haplotype sharing with ANP populations [24]. These differences in their genetic diversity may be the result of divergent colonization histories or of different migration abilities during the fragmentation process.

Our study provides evidence for a significant influence of the Mahajamba River on genetic differentiation but not as high as in their reddish sister species. Guschanski et al. [24] found that 82.7% of the molecular variance in the reddish mouse lemurs of northwestern Madagascar was explained by the Sofia and Mahajamba Rivers, while we found that the Mahajamba River explained only 32% of the molecular variation in *M murinus*. Note that at that time *M. ravelobensis *and *M. bongolavensis *specimen were still believed to form two clades within the same species. D-loop haplotypes from the western or eastern side of the river Mahajamba differed in more than 59 base pairs [24], compared to the minimum of 24 bp differences found in this study for *M. murinus*. A later study showed that the two reddish clades had already diverged into two separate species and that the river had probably promoted this speciation event [19]. This comparative evidence suggests that either the common ancestors of the reddish mouse lemur species colonized the IRS1 and IRS2 earlier than *M. murinus *or that *M. murinus *possesses larger migratory abilities that sustain gene flow between the IRSs (around the headwaters of the large rivers), counteracting genetic drift on both sides of the river. The latter explanation seems to be less likely, since the haplotypes of either side of the river have diverged completely, indicating that the river effectively prevents gene flow between the IRSs. A later arrival of *M. murinus *in the northwest compared to the ancestors of *M. ravelobensis *and *M. bongolavensis *would also explain why the grey mouse lemur clade did not yet undergo speciation in these two IRSs. Ultimately, this barrier function of the river may be sufficient so that populations in the two IRSs may eventually diverge into distinct species.

Taken together, the AMOVA and the network analyses suggest that the genetic differentiation in *M. murinus *within IRS1 and IRS2 reached levels comparable to the ones existing between the IRSs. This may indicate that the genetic lineages within the IRSs have diverged very early during the colonization process or that they result from several colonization events. These hypotheses are discussed in the next section.

### Signatures of a recent colonization history

Assuming a Pleistocene colonization scenario which is suggested by its recent date of divergence (about 1.4 Mya) from its southern sister species [21], we predicted that populations might still show signs of the initial spatial expansion into the IRS1. The occurrence of a range expansion was confirmed by our spatial analyses, as none of the samples significantly rejected the hypothesis of spatial expansion (*Model 1*). Surprisingly, most samples pointed to only one of the two different periods of expansion. Our modelling approach (*Model 2*) showed that two successive spatial expansions in small-sized populations generate a trimodal distribution similar to the observed distributions. It may be surprising that *Model 1 *is not significantly rejected even when there are three modes in the *mismatch distribution*. This can be explained by the fact that the test is always picking up the bigger of the two last modes as a signal of spatial expansion, without considering the smaller one (see Figure 2). The estimations for the age of the expansion (Tau values, Table 2) also correspond to this largest mode. The fact that only very few Tajima's *D *and no Fu's *Fs *values were significantly negative, may seem contradictory to the *mismatch distribution *results. This apparent contradiction may be explained by the lower statistical power of these statistics compared to *mismatch distributions *to detect spatial expansions when local demes exchange only few migrants (low *Nm*, which seems to be the case for *M. murinus*) as demonstrated theoretically [29] and empirically in the eastern tiger salamander [30]. This is also confirmed by our simulations of the double expansion where only 55.1% and 6.9% of the respective Tajima's *D *and Fu's *Fs *were negative and only 3.7% and 0.0% of those values were significant. Furthermore, a very recent simulation study by Städler et al. [31] confirmed that even when populations are undergoing a very important spatial expansion, Tajima's *D *can exhibit positive values (apparently indicating a bottleneck). Finally, these tests may also be affected by the recent fragmentation as noted above. Altogether, these analyses show the limitations of single statistics such as Tajima's *D *and Fu's *Fs *to detect demographic events when more than one such event has taken place [31,32].

The fact that we pick up the signals of spatial expansions in the *mismatch *analyses suggests that they took place relatively recently. It has already been suggested that the Malagasy forest habitats underwent severe changes during the Pleistocene due to climatic changes associated with the glacial and interglacial periods [3,18]. Together with the estimate of 1.4 My for the last common ancestor of *M. murinus *[21], it seems likely that *M. murinus *may have colonized the IRSs at its northern distribution range in the second half of the Pleistocene.

Based on the modes of the observed *mismatch distributions *expected under a spatial expansion model, it is possible to calculate rough estimates for the dates of these two successive spatial expansions. Assuming a constant mutation rate of 10^-6 ^per site per generation (one estimate used for a closely related lemur species [33]) and using the range of modes obtained for the samples, we can calculate the time of the expansions as tau*/(2×*mu*), where tau* is the mode of the *mismatch distribution *and *mu *is the mutation rate for the whole sequence. The first expansion was thereby estimated to have occurred between 26,500 to 33,500 years ago (modes between 24 and 30, Figure 2). The more recent expansion would then have taken place between 3,300 and 14,000 years ago (modes between three and 13). These estimations suggest that two recent expansions took place rather recently, one before and one after the last glacial maximum (LGM). Apparently, some populations have kept memory of the older and others of the more recent expansion, as was predicted by our simulations (Figure 4).

## Conclusions

Given the results of this study, the following scenario for the colonization of IRS1 by *M. murinus *is proposed: One ancestral population of *M. murinus *colonized the IRS1 probably before the last glacial maximum (LGM). *M. murinus *may then have retreated when forests contracted during the dry period associated with the LGM towards the persisting riverine forests along the Mahajamba and Betsiboka. It subsequently re-colonized the area (second wave of expansion) in a period of recent forest expansion following the LGM. This second spatial expansion is supported by the detection of a contact zone with high haplotype diversity, which parallels the events that were described in several other species as a result of postglacial expansions with subsequent admixture [34,35]. This contact zone is running through some central sites which exhibit haplotypes from three different haplotypes clusters. This interesting and new hypothesis deserves further investigations and needs to be formally tested in the future. In particular, the results obtained with our mtDNA dataset must be confirmed and complemented by other markers, such as nuclear STRs. Moreover, more refined simulation work taking into account different factors such as environmental heterogeneity or uncertainties about the mutation rate is needed to better define and date these processes. Indeed, no reliable estimate for the mutation rate is currently available for mouse lemurs. Using a mutation rate of 10^-7 ^[33], for example, would increase the age of the expansions by one order of magnitude (265,000 to 335,000 yrs for the older expansion and 33,000-140,000 yrs for the younger expansion). Although such a shift would still indicate a late Pleistocene dynamics, a reliable dating of these colonization processes would enable us to understand in more detail the evolution of Madagascar's extraordinary biodiversity and the underlying driving forces of diversification. So far, most studies have emphasized the ancient processes that triggered the many radiations that occurred on this island [36-38]. This is the first study detecting a potential genetic signature of late Pleistocene and maybe even postglacial biogeographic dynamics on this mini-continent. The further exploration of modern molecular modelling approaches may ultimately allow us to judge upon the relative importance of certain Pleistocene climate changes for the development of the biogeographic pattern that we can observe today. Mouse lemurs are an extremely suitable model to explore these processes across all Madagascar.

## Methods

### Study sites and samples

The study sites are located in northwestern Madagascar between two major rivers, the Betsiboka in the south and the Sofia in the north. The Mahajamba River divides this area into two IRSs (IRS1 and IRS2, Figure 1). IRS1 includes the Ankarafantsika National Park (ANP) as well as the isolated forest fragments S^te ^Marie, Tanambao and Mangatelo. IRS2 contains the fragments Tsinjomitondraka and Maroakata. The ANP (S 16°19', E 46°48') is one of the largest remaining forest areas in western Madagascar [12] and contains ten study sites (Figure 1). Two sites (Beronono, Bealana) are still connected with the ANP (Radespiel & Rakotondravony, unpublished data) and are thus considered hereafter as being part of the ANP group, even though they do not lie within the official borders of the ANP.

The forest fragments differed largely in size (2 km^2 ^- 40.8 km^2^) and were isolated from each other and the ANP by variable stretches of savannah. Euclidean distances between study sites ranged from 13 to 109 km with a maximum distance of 68 km between sites within the ANP. A total of 195 individual samples (♂: 106, ♀: 88, sex undetermined: 1), collected between the years 2000-2004, were available for this study. The number of individuals per site varied between 3 and 27 (Tab. 1). Samples were collected in form of ear biopsies (2-4 mm^2^) after capturing mouse lemurs systematically with Sherman Live traps as described in Olivieri et al. [19]. Double sampling of single individuals was prevented by marking all captured animals with an individual ear-cut pattern. Capturing and sampling protocols adhered to the legal requirements of Madagascar and were approved by CAFF/CORE, the "Direction Générale des Eaux et Forêts", and by ANGAP (required for the sites in the Ankarafantsika National Park).

### Molecular methods

DNA was isolated from the ear biopsies using a standard phenol/chloroform extraction [39]. A specified PCR for mitochondrial D-loop sequences was conducted using the universal mammalian control region primers L15997 (5'-CACCATTAGCACCCAAAGCT-3') and H16498 (5'-CCTGAAGTAGGAACCAGATG-3') [40]. The amplification of mtDNA followed the routines described in Guschanski et al. [24]. Purification of amplified PCR-products was performed with the Invitek purification kit (MSB^® ^Spin PCRapace) following the manufacturer's protocol. Sequencing was carried out by the company Macrogen (Seoul, South Korea, http://www.macrogen.com/eng/macrogen/macrogen_main.jsp). Sequences were analyzed with SeqMan II 6.00 (^© ^1989-2004 DNASTAR) and subsequently cut to a uniform length of 455 bp. The alignment of all sequences was constructed with the program Mega 4.0.

### Analysis of the genetic diversity and demographic history of the populations

The number of haplotypes and the haplotype and nucleotide diversities (*Nd*, [41]) were calculated with the program DnaSP 4.0 in order to determine the genetic variability within each population. Missing data or alignment gaps were not taken into account. Allelic richness (*AR*_(r)_), corrected for sample size variations, was calculated for each population with a sample size >3 from haplotype frequencies using the rarefaction method proposed by Petit et al. [42] with the software *RAREFAC*. We used a simple regression (GLM Type II, sum of squares) to test whether the number of haplotypes or haplotype diversity could be explained by sample size. Furthermore, a Mann-Whitney U-Test was used to determine if there was a difference in genetic diversity (*Hd*, *AR*_(r) _and *Nd*) between the ANP samples and the samples from isolated forest fragments. All tests were done using the STATISTICA 5.5 software.

Tajima's *D *[43] und Fu's *Fs *[44] are known to be sensitive to departures from mutation-drift equilibrium due to population size changes (expansions, bottlenecks) and selection. They were thus computed for all samples using the Arlequin v.3 software [45]. Following the recommendations in the manual, the Fu's *Fs*-statistic was regarded as significant with a p-value lower than 0.02 instead of 0.05. Negative values of both statistics point towards population growth and/or positive selection, whereas positive Tajima's *D *values indicate bottlenecks and/or balancing selection.

In addition to these tests, *mismatch distributions *were constructed for polymorphic samples with a sample size of at least 10 individuals. A *mismatch distribution *is the distribution of the number of nucleotide mismatches between all pairs of DNA sequences belonging to a population sample. The shape of the *mismatch distribution *has been shown to be influenced by past demographic events such as expansions and bottlenecks. The distribution is usually bell-shape in populations having increased demographically in the past [46], while it shows one or two modes in populations having passed through a range expansion, depending on the population density and the amount of migrants exchanged with neighbouring populations [29].

Using the software Arlequin v.3, it is possible to formally test if a *mismatch distribution *rejects statistically the null hypothesis of one spatial expansion, which we call *Model 1 *hereafter. In this model it is assumed that a population expanded spatially from one deme into a larger area divided in same-sized demes interconnected by gene flow. We estimated also estimated *Tau*, the time of the expansion (in mutation units, Tau = 2 *Tμ*).

**Figure 8:**
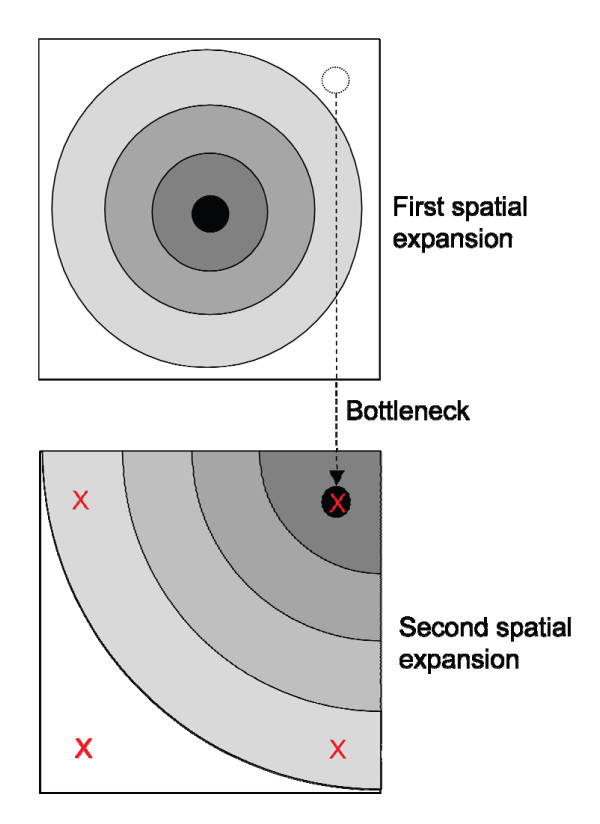
**Summary of the dynamics of the simulations of two successive spatial expansions**. A first spatial expansion (upper graph) is simulated in a subdivided population of 2,500 demes arranged as a two-dimensional stepping-stone (50 × 50 demes). Each shade of gray denotes the limit of the area of occupied demes at different steps of the progression of the expansion (darker gray shows the first steps while lighter gray shows the more recent steps). At the end of the first spatial expansion a bottleneck is simulated in taking 50 individuals from a peripheral deme from the first grid to start a second spatial expansion (lower graph) in an unoccupied grid. Red crosses show sampling locations at the end of the second spatial expansion.

Because the *mismatch distribution *of all the samples from IRS 1 taken together shows three different modes, which is unexpected under *Model 1*, we investigated a second model (*Model 2)*, which consists of two successive spatial range expansions interrupted by a (moderate) bottleneck (see below). Since no formalized test is currently available for this model, we simulated data according to *Model 2 *and visually compared the *mismatch distributions *generated to those observed. Simulations were carried out using the modified version of the SPLATCHE software [47] described in Currat & Excoffier [48]. Basically, the simulation framework consists in two grids of 2,500 demes arranged in stepping-stone pattern as described in Ray et al. [29]. Instead of simulating only one spatial expansion, two successive spatial expansions were simulated following the methodology detailed in Currat [49]. The first expansion is simulated in one grid of demes from a central deme. After the first grid has been fully colonized, 50 individuals, taken from a peripheral deme, start a new spatial expansion in the second grid (see Figure 8). Using the coalescent approach, sixty DNA sequences of 450 bp are then drawn from four different peripheral demes (two demes with 20 sequences each and two demes with 10 sequences each). The chosen demes vary in size and are located in the periphery to account for the diverse sampling locations and sizes in the *M. murinus *dataset and also because the IRSs studied here are more likely to represent the periphery than the centre of the expansion. This procedure allows us to simulate the genetic diversity of mitochondrial sequences that underwent an old spatial expansion, then passed through a bottleneck, corresponding to the reduction in size of viable habitat and finally through a more recent spatial expansion into an empty territory. Parameters which correspond approximately to those estimated from our samples were chosen: the older expansion occurring about 30 mutational units of time in the past and the more recent one 10 mutational units of time ago (see the *mismatch distribution *results). We used a mutation rate of 10^-6 ^per site and per generation [33], a carrying capacity *K *equal to 50 for all demes, a growth rate of 0.2 and a generation time of one year. Using a migration rate of 0.1, the effective number of migrants exchanged between demes at equilibrium (*Nm*) is equal to 5.

### Analysis of genetic structure

Genetic differentiation between populations was measured using Φ_ST_. The model of Tamura & Nei [50] was chosen with the gamma correction factor of 0.6354, as determined with the program Modeltest 3.5 mac [51] on the basis of the Akaike Information Criterion (AIC). The significance of these values was estimated with 1000 permutations as implemented in Arlequin v.3. A Mantel test [52] was performed with XLSTAT v. 2007.7 in order to investigate the relationship between geographic and genetic distances (Φ_ST_-values). The statistical significance was determined by means of 10,000 permutations.

An Analysis of Molecular Variance (AMOVA) was carried out in order to detect genetic structure generated by the river Mahajamba, separating the two IRSs. For this analysis, samples were divided into two groups corresponding to IRS1 und IRS2, which makes the results directly comparable to study of Guschanski et al. [24] on the other species of mouse lemurs in the area. The input file was constructed with Seqtrans 1.1 (Dr. S. M. Funk) and the AMOVA was performed with Arlequin v.3 [45]. Two populations with very small sample sizes (*n *< 5: Bealana, Komandria) were excluded from this analysis.

The influence of the forest fragmentation on genetic differentiation was tested for the populations in IRS1 by comparing the pairwise Φ_ST _values obtained between populations separated by stretches of savannah (ANP group vs. fragments and between fragments) and those obtained for pairs of populations separated by a "continuous" forest habitat, i.e. within the ANP group including Beronono and Bealana. Since geographic distance is a confounding factor that may inflate "savannah" distances, we only compared pairwise Φ_ST _values for geographic distances lower than the highest one found within the ANP. Consequently, of the three fragments available, Tanambao was excluded from the analysis due to its large distance from the ANP. We regressed Φ_ST _values against geographical distances within the ANP and then compared the residuals obtained with those corresponding to pairs involving a savannah crossing. If the savannah generated an increase in genetic differentiation we expect that the savannah residuals should be more positive than the ANP residuals (i.e. the Φ_ST _values would be higher for a similar geographic distance). The difference between the two sets of residuals was tested using a permutation approach (10,000 permutations). We also tested for a correlation between the residuals and geographical distances. These computations were performed with *R *[53] partly with available and partly with self-written scripts.

Finally, for the analysis of genetic structure and haplotype sharing, a haplotype network was constructed with the programme TCS 1.21 [54]. Clusters of closely related haplotypes (connected by less than 10 mutation steps) were identified and mapped geographically.

## Authors' contributions

NS sequenced the samples, performed the alignment, contributed to some analyses and was involved in drafting the manuscript. LC participated in the design of the study, contributed some statistical analyses and was involved in the interpretation and drafting of the manuscript. MC contributed some statistical analyses and was involved in the interpretation and drafting of the manuscript. UR conceived of the study, participated in its design and in data interpretation and drafted the manuscript. All authors read and approved the final manuscript.

## Authors' informations

NS was an undergraduate Biology student in the lab of UR when conducting this population genetics study. LC is interested in understanding how genetic data can be used to reconstruct the demographic history of species with an emphasis on endangered species and on humans. MC leads a research program on population genetics, with a focus on the genetic consequences of past demographic events and natural selection. UR is co-leading a long-term project on the evolution and behavioral ecology of nocturnal lemurs in northwestern Madagascar which is conducted by members of the Institute of Zoology, University of Veterinary Medicine Hannover.

## Supplementary Material

Additional file 1 **Table S1 - Pairwise Θ_ST_-values between all study sites (above diagonal) and their significance (below diagonal)**. ANP: sites in the Ankarafantsika National Park, IFFs: Isolated forest fragments, ****: p < 0.0001, **: p < 0.01, *: p < 0.05, n.s.: not significant. For abbreviations of study sites see Table 1. Click here for file
