# Supplementary material for: Signals of recent spatial expansions in the grey mouse lemur (Microcebus murinus)
Source: BMC Evol Biol. 2010 Apr 22;10:105. doi: 10.1186/1471-2148-10-105 (PMC2875232; doi:10.1186/1471-2148-10-105)
Supplement: Additional file 1 — Table S1 - Pairwise ΘST-values between all study sites (above diagonal) and their significance (below diagonal). ANP: sites in the Ankarafantsika National Park, IFFs: Isolated forest fragments, ****: p < 0.0001, **: p < 0.01, *: p < 0.05, n.s.: not significant. For abbreviations of study sites see Table 1. [file 1471-2148-10-105-S1.DOC]

**Additional file 1 – Table S1: Pairwise Ф_ST_-values between all study sites (above diagonal) and their significance (below diagonal).**

|  | **IRS** | **1** | **1** | **1** | **1** | **1** | **1** | **1** | **1** | **1** | **1** | **1** | **1** | **1** | **2** | **2** |
| --- | --- | --- | --- | --- | --- | --- | --- | --- | --- | --- | --- | --- | --- | --- | --- | --- |
|  |  | **ANP** | **ANP** | **ANP** | **ANP** | **ANP** | **ANP** | **ANP** | **ANP** | **ANP** | **ANP** | **IFF** | **IFF** | **IFF** | **IFF** | **IFF** |
|  | **site** | **Bev** | **Anko** | **kely** | **fom** | **bodi** | **Bero** | **Ando** | **JBA** | **Bea** | **Kom** | **StM** | **telo** | **Tan** | **ata** | **Tsin** |
|  | **Bev** |  | 0.2897 | 0.2880 | 0.3285 | 0.8468 | 0.2693 | 0.3361 | 0.6993 | 0.7720 | 0.8158 | 0.4315 | 0.7620 | 0.7843 | 0.9189 | 0.8839 |
|  | **Anko** | **** |  | 0.3236 | 0.3629 | 0.7554 | 0.3103 | 0.2466 | 0.6530 | 0.6813 | 0.7036 | 0.4428 | 0.6833 | 0.7405 | 0.8385 | 0.7975 |
|  | **kely** | **** | **** |  | 0.1467 | 0.5799 | 0.2546 | 0.2746 | 0.5077 | 0.5092 | 0.5410 | -0.0307 | 0.5738 | 0.4582 | 0.7862 | 0.7064 |
| **ANP** | **fom** | **** | **** | **** |  | 0.2900 | 0.2906 | 0.2521 | 0.1572 | 0.1193 | 0.1757 | 0.2056 | 0.5571 | 0.4315 | 0.6658 | 0.6148 |
| **IRS 1** | **bodi** | **** | **** | **** | **** |  | 0.9105 | 0.7449 | 0.3369 | 0.7064 | 1.0000 | 0.6526 | 0.9951 | 0.8542 | 1.0000 | 0.9723 |
|  | **Bero** | * | **** | ** | ** | **** |  | 0.2854 | 0.7026 | 0.7964 | 0.8729 | 0.3738 | 0.9026 | 0.8133 | 0.9708 | 0.9147 |
|  | **Ando** | **** | **** | **** | **** | **** | n.s. |  | 0.6113 | 0.6350 | 0.6749 | 0.3877 | 0.6932 | 0.7180 | 0.8567 | 0.8097 |
|  | **JBA** | **** | **** | **** | ** | **** | **** | **** |  | -0.0226 | 0.1450 | 0.5801 | 0.8835 | 0.7539 | 0.8989 | 0.8549 |
|  | **Bea** | **** | **** | **** | n.s. | * | * | **** | n.s. |  | -0.0165 | 0.5766 | 0.9753 | 0.7992 | 0.9782 | 0.9324 |
|  | **Kom** | **** | **** | **** | n.s. | ** | ** | **** | n.s. | n.s. |  | 0.6126 | 0.9948 | 0.8390 | 1.0000 | 0.9632 |
| **IRS 1** | **StM** | ** | **** | n.s. | **** | **** | * | **** | **** | **** | ** |  | 0.6918 | 0.3885 | 0.8291 | 0.7337 |
| **IFFs** | **telo** | **** | **** | **** | **** | **** | **** | **** | **** | **** | **** | **** |  | 0.9301 | 0.9971 | 0.9831 |
|  | **Tan** | **** | **** | **** | **** | **** | **** | **** | **** | ** | **** | **** | **** |  | 0.9324 | 0.8911 |
| **IRS 2** | **ata** | **** | **** | **** | **** | **** | **** | **** | **** | **** | **** | **** | **** | **** |  | 0.9850 |
| **IFFs** | **Tsin** | **** | **** | **** | **** | **** | **** | **** | **** | ** | **** | **** | **** | **** | **** |  |

ANP: sites in the Ankarafantsika National Park, IFF(s): Isolated forest fragment(s), ****:p < 0.0001, **: p< 0.01, *: p < 0.05, n.s.: not significant. For abbreviations of study sites see Table 1.
